# Development and Validation of Kompetitive Allele-Specific Polymerase Chain Reaction Markers for Seed Protein Content in Soybean

**DOI:** 10.3390/plants13243485

**Published:** 2024-12-13

**Authors:** Shuangzhe Li, Chenyijun Guo, Xuezhen Feng, Jing Wang, Wenjing Pan, Chang Xu, Siming Wei, Xue Han, Mingliang Yang, Qingshan Chen, Jinxing Wang, Limin Hu, Zhaoming Qi

**Affiliations:** 1 National Key Laboratory of Smart Farm Technology and System, Key Laboratory of Soybean Biology in Chinese Ministry of Education, College of Agriculture, Northeast Agricultural University, Harbin 150030, China; shuangzheli@163.com (S.L.); chenyijunguo@163.com (C.G.); fengxz_1998@163.com (X.F.); wangjing_3660@163.com (J.W.); xuchang@neau.edu.cn (C.X.); wsm20016@126.com (S.W.); hanxue1860@126.com (X.H.); yml5418@126.com (M.Y.); qshchen@126.com (Q.C.); 2 Suihua Branch of Heilongjiang Academy of Agricultural Sciences, Suihua 152052, China; wadrjwpp@163.com (W.P.); wjxsuihua@126.com (J.W.)

**Keywords:** *Glycine max*, seed protein content, KASP, marker-assisted selection

## Abstract

Seed protein content is a critical trait in soybean breeding, as it provides a primary source of high-quality protein for both human consumption and animal feed. This study aimed to enhance molecular marker-assisted selection for high-protein soybean varieties by developing Kompetitive Allele-Specific Polymerase Chain Reaction (KASP) markers targeted at loci associated with seed protein content. Nineteen markers with high genotyping efficacy were identified through screening. Utilizing SN76 (a high-protein line) as the male parent and SN49 and DS1 (both low-protein lines) as female parents, 484 F_6_ generation individuals from these hybrid combinations were selected to validate the predictive accuracy of the 19 KASP markers. Notably, KASP-Pro-1, KASP-Pro-2, and KASP-Pro-3 effectively distinguished genotypes associated with high and low protein content, with prediction accuracies of 68.4%, 75.0%, and 83.3%, respectively. These results underscore the reliability and practical utility of the selected molecular markers, which are located within the genes *Glyma.03G219900*, *Glyma.14G119000*, and *Glyma.17G074400*, respectively. Haplotype analysis and gene pyramiding indicate that these three genes may influence seed protein content. Consequently, these KASP markers can be effectively integrated into genetic and genomic research on soybean seed protein content as well as into marker-assisted breeding.

## 1. Introduction

Soybean (*Glycine max* L. Merrill) is a vital global crop that serves as a foundation for food, oil, and forage industries. Its seeds are rich in protein, forming about 40% of their content, and in oil, containing about 20% [1,2]. With the world’s population projected to reach 9.7 billion by 2050 [3], there will be significant demands on the food supply, particularly for plant-based proteins. Globally, soybeans contribute about 70% of protein in animal feed and plant-based foods, which can help meet the calorie and protein needs of the growing global population [4,5,6,7]. Therefore, the development of soybean cultivars with higher protein content presents an effective strategy to meet this escalating demand.

Soybean seed storage proteins are categorized into 2S, 7S, 11S, and 15S proteins based on their sedimentation coefficients [8]. β-Conglycinin (7S) and glycinin (11S) are the major components of soybean protein, accounting for 70–80% of total protein [9]. Glycinin is composed of six subunits, each comprising an acidic and a basic polypeptide chain, which are covalently linked by disulfide bonds [10]. β-Conglycinin is made up of three distinct subunits: α, α’, and β, with isoelectric points of 4.90, 5.18, and 5.66–6.00, respectively [11]. While the 11S proteins are primarily storage proteins, the 7S proteins have been implicated in the regulation of plant growth and development [9]. Seed protein content is a complex quantitative trait, controlled by an interplay of multiple genes and regulatory elements [12]. Over recent decades, the genetic landscape of this trait has been mapped in considerable detail, resulting in the documentation of as many as 305 quantitative trait loci (QTLs) associated with soybean protein (SoyBase, https://www.soybase.org, accessed on 2 September 2024). Advances in sequencing technology, complemented by the application of novel molecular markers, have propelled the identification of several key genes that govern soybean seed protein content. These key genes include *GmSWEET39*, *GmSWEET10a*, *POWR1*, *GmJAZ3*, and *GmST05* [13,14,15,16,17]. The discovery and characterization of these genes offer valuable targets for enhancing soybean protein content.

Timely and precise evaluation of traits is indispensable for genetic screening, genomic selection, and gene identification within large-scale soybean breeding programs, where the rapid assessment of extensive plant populations is necessary [18,19]. In soybean breeding, the total protein and oil content are conventionally measured with near-infrared (NIR) spectroscopy, a method known for its reliability [20]. Nonetheless, this approach faces challenges, including the requirement for mature sampling and the prolonged time for phenotype data acquisition, which could limit the breeding cycle. Molecular markers offer an effective and efficient approach for crop breeding due to their capacity for high-throughput genotyping and precision selection. These attributes contribute to a reduction in the duration of breeding cycles and an increase in overall breeding efficiency [21].

In molecular breeding, a variety of markers have been developed, including restriction fragment length polymorphism (RFLP), random amplified polymorphic DNA (RAPD), simple sequence repeat (SSR), and single-nucleotide polymorphisms (SNPs) [22,23,24,25,26,27]. Over the past two decades, SNPs have risen to prominence as the markers of choice in molecular breeding due to their genomic abundance, ease of scoring, amenability to high-throughput genotyping, and a relatively low incidence of genotyping errors [28]. Among these molecular markers, Kompetitive Allele-Specific PCR (KASP) has distinguished itself as the leading method for SNP genotyping [29]. The KASP marker has risen to prominence due to its high throughput, minimal error rate, cost-effectiveness, and its ability to detect both homozygous and heterozygous genotypes [30]. The KASP genotyping technique, which detects fluorescence signals during the PCR amplification phase, facilitates high-throughput and highly accurate genotyping. These attributes render KASP technology particularly well suited for applications in crop breeding and genetic trait enhancement [31,32]. In addition, KASP technology is instrumental in QTL mapping, gene identification, resource characterization, and other critical applications, with successful implementation in a spectrum of crops, including rice, maize, wheat, broad bean, and so on [33,34,35,36,37,38,39,40]. Therefore, the development of efficient KASP markers for the identification of soybean seed protein content is important.

The KASP marker system is extensively utilized across a range of crops [41,42,43,44]. It has been effectively integrated into the selection of various traits, including wheat resistance to Fusarium head blight [45], cold tolerance in maize [46], and aromatic quality and seed anaerobic germination in rice [47,48], showcasing its versatility across diverse genetic contexts. In soybean breeding, KASP markers have been instrumental for gene identification, molecular-assisted selection, and genetic resource characterization [49,50]. To date, KASP markers have been developed for multiple soybean traits, encompassing salt tolerance, pod number, leaf morphology, pod setting patterns, tolerance to pod drop, and trypsin inhibitor levels [49,51,52,53]. Nonetheless, there is a shortage of markers dedicated to selecting for soybean seed protein content. In this study, we developed three KASP markers related to seed protein content and validated their genotype typing and phenotype prediction accuracy. Further analysis was conducted on potential candidate genes for seed protein content. These KASP markers could offer valuable information for the molecular breeding of soybeans with high protein content.

## 2. Results

### 2.1. Development of KASP Makers

In this study, we have collected a comprehensive list of candidate genes related to soybean seed content, including 102 genes previously identified in our lab (Table S1).

**Figure 1:**
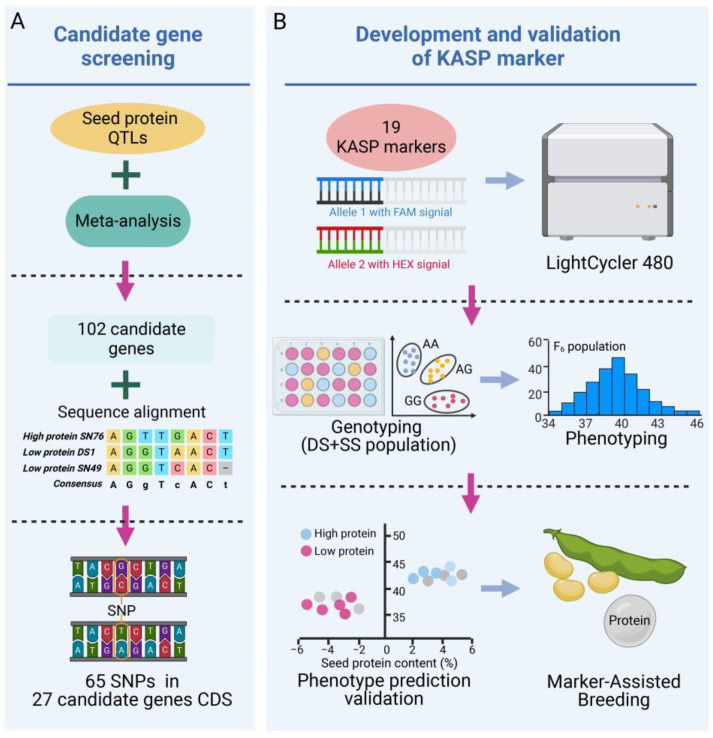
Flowchart for KASP marker development, genotyping, and phenotype prediction validation based on candidate genes for soybean seed protein content. (**A**) Screening of candidate genes for seed protein content. (**B**) Development and validation of KASP markers.

By conducting a collinear relationship analysis and meta-analysis on 231 known QTLs for seed protein content, important collinear segments and meta-QTLs were obtained, and 102 candidate genes were selected based on transcriptome data [54,55,56]. Gene annotations suggest that these candidates may play important roles in fundamental biological processes, including fatty acid and protein synthesis and plant growth and development. Multiple sequence alignments of the entire gene sequence of these 102 candidate genes were conducted using the sequences from the high-protein-content parent SN76 and the low-protein-content parents SN49 and DS1. For KASP markers, we selected the SNPs in the coding sequence (CDS) regions in these 102 candidate genes (Figure 1). Following the screening process, a total of 65 SNP sites were pinpointed within the 27 candidate genes (Table S2).

For each identified SNP site, flanking sequences of 50 base pairs upstream and downstream were extracted. Primers for KASP assays were designed, and they detected the genotyping effect using the high-protein-content parent SN76 and the low-protein-content parents SN49 and DS1. This rigorous process led to the selection of 19 pairs of KASP markers that demonstrated high genotyping efficacy (Figure 1). These markers are mapped across eight distinct chromosomes: Chr02 and Chr07 each present four SNPs, Chr03, Chr12, and Chr14 harbor two SNPs each, Chr15 and Chr19 feature a single SNP, and Chr17 exhibits three SNPs (Table 1).

### 2.2. Validation of KASP Markers

**Figure 2:**
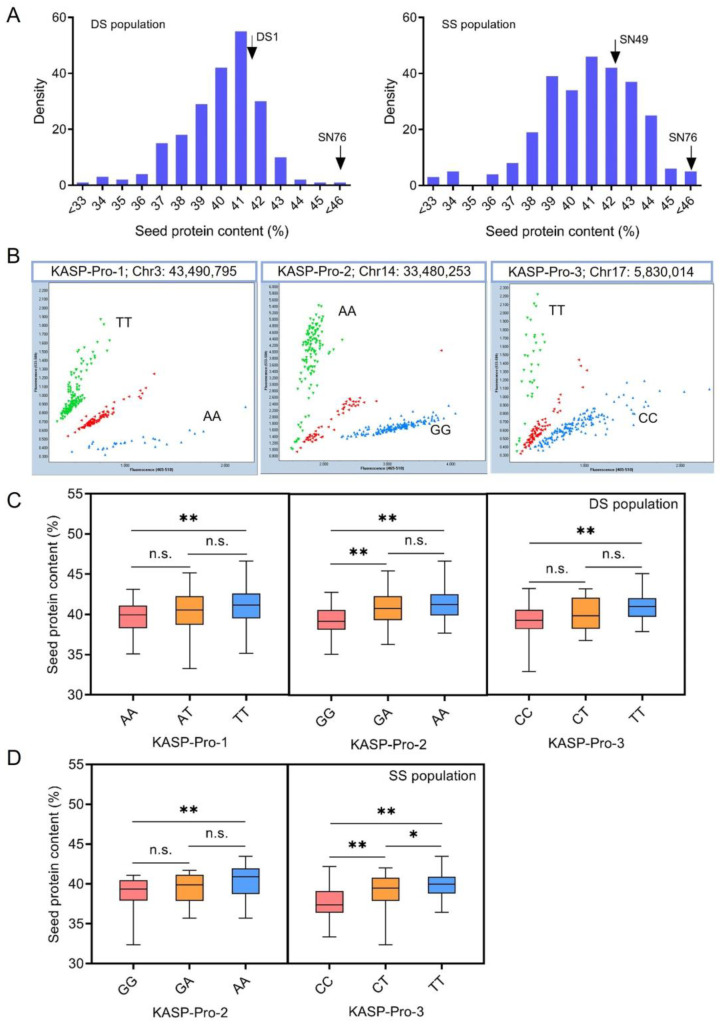
Allelic discrimination of hybrid populations using the KASP-Pro-1, KASP-Pro-2, and KASP-Pro-3 markers. (**A**) A histogram of seed protein phenotype distribution in the DS and SS populations. The black arrows indicate parental phenotype. (**B**) Genotyping using three KASP markers in the DS and SS populations. Each dot corresponds to an individual test; the green and blue dots represent homozygous genotypes, and the red dots represent heterozygotes. (**C**) A phenotypic comparison of genotyping using three KASP markers in the DS population. (**D**) A phenotypic comparison of genotyping using KASP-Pro-2 and KASP-Pro-3 in the SS population. *, **, and n.s. represent significant differences at *p* < 0.05, significant differences at *p* < 0.01, and no significance, respectively.

To validate the KASP markers in hybrid populations, two hybrid populations of F_6_ generation were developed using the high-protein soybean variety SN76 and the low-protein varieties DS1 and SN49. They had significant differences in protein content and amino acid composition (Table S3). We genotyped the lines of these populations with 19 KASP markers and determined the seed protein content in the 212 lines from DS1 × SN76 (DS population) and 272 lines from SN49 × SN76 (SS population) (Figure 1 and Figure 2A). The seed protein content of the two populations showed abundant phenotypic variation (Table S4). Factors such as genotype environment interaction and selection pressure may have resulted in seed protein content in multiple samples being lower than the parental phenotype [57]. In the scatter plot, the hybrid lines shown by the green dots have a high-protein homozygous allele of SN76; those shown by the blue dots have a low-protein homozygous allele of DS1 or SN49; and those shown by the red dots have a heterozygous genotype (Figure 2B, Figures S1 and S2).

**Figure 3:**
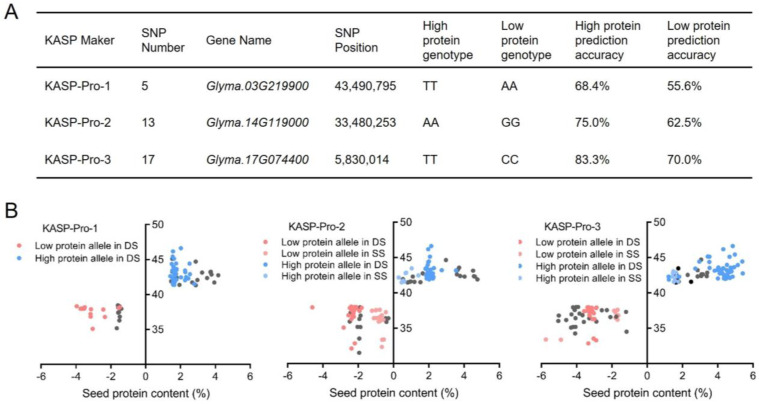
Statistics and graphical display of phenotype prediction accuracy of seed protein content with three KASP markers. (**A**) Statistics of phenotype prediction accuracy of seed protein content with three KASP markers. (**B**) Graphical display of phenotype prediction accuracy of seed protein content with three KASP markers.

**Figure 4:**
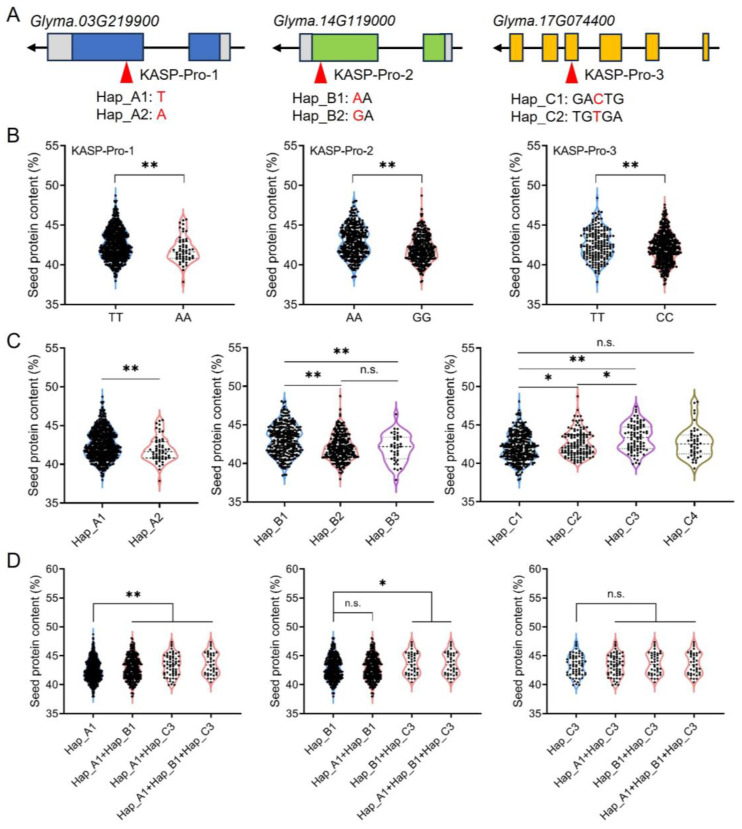
Haplotype analysis and gene pyramiding of *Glyma.03g219900*, *Glyma.14g119000*, and *Glyma.17g074400*. (**A**) Schematic representation of gene structure and KASP maker positions. (**B**) three KASP markers for genotype and phenotype classification of germplasm resources. (**C**) Haplotype analysis of three candidate genes in germplasm resources. (**D**) Changes in seed protein content among different haplotype pyramidings. * represents significant differences at *p* < 0.05, and ** represents significant differences at *p* < 0.01, n.s. represent no significance.

By integrating the genotyping data of 19 KASP markers with phenotypic measurements of seed protein content in the DS and SS populations, three KASP markers were identified with notable predictive accuracy (Figure 2C,D). Specifically, KASP-Pro-1, located on chromosome 3 at position 43,490,795, was correlated with TT genotypes in high-protein materials, at a conformity rate of 68.4%, and with AA genotypes in low-protein materials, at a rate of 55.6%. KASP-Pro-2, on chromosome 14 at position 33,480,253, was associated with AA genotypes in high-protein materials, exhibiting a conformity rate of 75.0%, and with GG genotypes in low-protein materials, exhibiting a rate of 62.5%. KASP-Pro-3, located on chromosome 17 at position 5,830,014, was linked to TT genotypes in high-protein materials, demonstrating a conformity rate of 83.3%, and to CC genotypes in low-protein materials, demonstrating a rate of 70.0%. The comprehensive predictive accuracy was calculated by averaging the accuracies from both high- and low-protein predictions. Therefore, these three KASP markers demonstrated prediction accuracies of 68.4%, 75.0%, and 83.3%, respectively, when distinguishing between high- and low-protein genotypes within the hybrid population (Figure 3). These three SNPs are located in the coding region of *Glyma.03G219900*, *Glyma.14G119000*, and *Glyma.17G074400* genes, respectively (Figure 4A). 

### 2.3. Haplotype Analysis of Candidate Genes

To further evaluate the correlation between these three key candidate genes and soybean seed protein content, a comprehensive phenotype and haplotype analysis was conducted on these genes and their corresponding KASP markers using 643 accessions. These 643 accessions have been resequenced, and the seed protein content shows abundant phenotypic variation (Table S5). Employing three KASP markers for the identification of accession genotypes can effectively differentiate between high-protein and low-protein phenotypes (Figure 4B). The results of the haplotype analysis for *Glyma.03G219900*, *Glyma.14G119000*, and *Glyma.17G074400* are illustrated in Figure 4C. Two haplotypes were identified for *Glyma.03G219900*, including the high-protein superior haplotype Hap_A1 and the low-protein haplotype Hap_A2. Furthermore, Hap_A1 demonstrates an average seed protein content of 42.6%, significantly surpassing the 41.9% observed for Hap_A2. *Glyma.14G119000* can be categorized into three distinct haplotypes, with Hap_B1 recognized as the high-protein haplotype and Hap_B2 and Hap_B3 recognized as the low-protein haplotype. Hap_B1 showed an average seed protein content of 43.1%, which is significantly higher than the 42.1% and 42.0% observed for Hap_B2 and Hap_B3. *Glyma.17G074400* is characterized by four haplotypes, with Hap_C3 being the high-protein haplotype with the highest seed protein content of 43.4%. Hap_C2 is also a high-protein dominant haplotype, with a protein content of 42.6%. In contrast, Hap_C1 is a low-protein haplotype, featuring a seed protein content of 42.1% (Figure 4C).

### 2.4. Pyramiding Effect Analysis of Candidate Genes

To further determine if the superior haplotypes in high-protein materials have a synergistic effect, an in-depth analysis of the aggregation effect of three candidate genes was performed. Among the 643 soybean accessions, the haplotype distribution was characterized by the following counts: 542 for Hap_A1, 363 for Hap_B1, 65 for Hap_C3, 325 for the Hap_A1/Hap_B1 combination, 61 for Hap_A1/Hap_C3, 43 for Hap_B1/Hap_C3, and 42 for the triple combination Hap_A1/Hap_B1/Hap_C3. We identified various combinations of these excellent haplotypes, including Hap_A1/Hap_B1, Hap_A1/Hap_C3, and Hap_A1/Hap_B1/Hap_C3. The germplasm with these haplotype combinations exhibited average seed protein contents of 43.0%, 43.6%, and 43.6%, respectively, all of which were significantly higher than the 42.6% observed in germplasm with only the Hap_A1 haplotype. This indicates that the co-occurrence of Hap_B1 and Hap_C3 haplotypes significantly boost the seed protein content in germplasm carrying Hap_A1. For the Hap_B1 haplotype, the protein content of the aggregated Hap_A1/Hap_B1 material was nearly identical at 43.0%. The average seed protein content for the Hap_B1/Hap_C3 and Hap_A1/Hap_B1/Hap_C3 combinations was higher than that of Hap_B3 alone although not statistically significant. For Hap_C3, as well as the combinations Hap_A1/Hap_C3, Hap_B1/Hap_C3, and Hap_A1/Hap_B1/Hap_C3, the aggregation types resulted in comparable seed protein contents (Figure 4D).

## 3. Discussion

In soybean, KASP markers have been developed for structural variants linked to *GmCHX1* and were validated for having a strong correlation between genotype and salinity tolerance [49]. KASP markers were developed for two candidate genes, PI594891 and PI594774, which are implicated in resistance to *Cercospora cinerea*, to evaluate genotype–phenotype associations within populations [58]. Additionally, KASP markers (GSM151) were created to screen for resistance to *Cysticercus elegans* in soybean varieties [59]. Recently, 21 KASP markers were developed, corresponding to reported functional loci of important traits in soybean, and could be used for accurate genotyping [52]. Despite the widespread application of KASP markers, there is a scarcity of reports on their development for soybean seed protein content. This study collected 102 genes previously suggested to regulate seed protein content (Table S1). These candidate genes for KASP marker development were based on the seed protein content meta-QTLs analysis currently conducted in our laboratory, which includes 231 seed-protein-content-related QTLs published between 1992 and 2015 [54,55,56]. By employing resequencing data from the high-protein cultivar SN76 and two low-protein cultivars, SN49 and DS1, these genes were extracted and compared to pinpoint candidate genes that influence seed protein content. Among them, 27 genes displayed missense mutations in their coding regions, leading to the identification of 67 SNPs for the development of subsequent KASP markers (Table S2). After rigorous screening, 19 pairs of KASP markers exhibiting high genotyping efficacy were identified (Table 1). Following the validation of genotyping efficiency and phenotypic prediction in hybrid populations, three highly efficient KASP markers were ultimately selected for use (Figure 1).

The predictive efficiency of KASP markers for target phenotypes is critically important. Prior research has successfully developed KASP markers associated with pod setting habits, leaf morphology, multiple pod formations, and pod dehiscence, with phenotypic prediction accuracies ranging from 84.0% to 94.8% [53]. In the present study, 19 KASP markers showed effective genotyping outcomes (Figure 1, Figures S1 and S2). Moreover, the phenotypic prediction efficiency was further validated using the F_6_ segregating population, where only three markers displayed high accuracy in forecasting seed protein content (Figure 3). The concordance rates for high-protein materials were 68.4% for KASP-Pro-1, 75.0% for KASP-Pro-2, and 83.3% for KASP-Pro-3; whereas for low-protein materials, these rates were 55.6%, 62.5%, and 70.0%, respectively (Figure 3). The phenotypic prediction accuracy for these three newly developed KASP markers was below 84.0%, likely due to the fact that pod setting habits, leaf shape, and multi-pod traits are traits controlled by major genes and are minimally influenced by environmental factors [60,61]. In contrast, seed protein content is a complex quantitative trait regulated by multiple genes and subject to the influence of environmental factors, leading to diverse phenotypic prediction accuracies. For example, KASP markers developed for flowering traits have also shown inconsistencies between genotype identification and phenotypic expression [62]. In this study, inconsistencies between genotype and phenotype were also observed in the validation of three KASP markers, which affected the accuracy of prediction.

Seed protein content is closely related to seed yield and yield-related traits. In the breeding of varieties with high seed protein content, yield components should also be considered [63]. In this study, the QTLs containing the three genes not only affect seed protein content but also affect seed yield and yield-related traits (Table S6). *Glyma.03g219900* is located in QTLs, which affect multiple traits such as seed yield, seed oil content, seed thickness, and seed length [64,65,66,67,68,69]. *Glyma.14g119000* is located in QTLs that affect seed yield, seed oil content, and seed width [64,68,70,71,72]. The QTLs containing *Glyma.17g074400* affect seed oil content, seed weight, and shoot weight [73,74,75]. The phenotyping of complex quantitative traits must be conducted in at least three independent test environments [76,77]. These three QTLs were repeatedly identified in multiple hybrid combinations, environments, and years. Therefore, while screening for high protein content, the three KASP markers may also have selected seed yield and yield-related traits. In this study, the validation of the prediction accuracy of KASP markers was mainly based on the phenotype of seed protein content. The screening effect on other yield traits needs further exploration and evaluation.

Three novel KASP markers have been identified within the genes *Glyma.03g219900*, *Glyma.14g119000*, and *Glyma.17g074400*, respectively (Figure 4A). Their high accuracy in predicting phenotypic traits renders these genes important candidates influencing the protein content of soybean seeds. *Glyma.03g219900* encodes a DELLA subfamily member and is orthologous to *RGL3* (*AT5G17490*) in Arabidopsis, which acts as a negative regulator of GA signaling and as a coactivator of ABI3 to promote seed storage protein biosynthesis during the seed maturation stage [78]. *Glyma.14g119000* encodes a MYB transcription factor, known in plants to regulate processes such as morphogenesis [79] and the synthesis of flavonoids [80]. The role of flavonoids in modulating seed protein and fatty acid accumulation has been highlighted in recent studies [81]. *Glyma.17g074400* is responsible for encoding omega-6 fatty acid desaturase enzyme, essential for the synthesis of unsaturated fatty acids. Specific FAD genes, such as *GmFAD3* and *GmFAD3-2a*, have been shown to affect soybean seed size and oil content [82,83]. Collectively, these candidate genes were suspected of playing a substantial role in the synthesis and accumulation of protein and oil in soybean seeds.

Haplotype analysis offers valuable insights into the genetic combinations and patterns across different loci within specific chromosomal regions, which are instrumental in uncovering beneficial allelic variations for crop breeding [84]. To further clarify the influence of three candidate genes on seed protein content, a haplotype analysis was performed using 643 accessions, complemented by phenotypic identification (Figure 4C). Each of the three candidate genes possesses a dominant haplotype correlated with high protein content. For *Glyma.03g219900*, the high-protein haplotype, designated Hap_A1, exhibited an average seed protein content of 42.6% and accounted for 84.3% in germplasm resources. The high-protein haplotype of *Glyma.14g119000*, referred to as Hap_B1, had an average seed protein content of 43.1% and accounted for 56.5% in germplasm resources. The superior haplotype of *Glyma.17g074400*, known as Hap_C3, resulted in an average seed protein content of 43.3%, present in 10.1% of the occurrences. Furthermore, the pyramiding effect of three candidate genes was examined. In the case of Hap_A1 germplasm, it sustained a high protein content of 42.6%. Meanwhile, the combined presence of Hap_B1 and Hap_C3 significantly elevated the seed protein content. Concurrently, the various aggregation types of these three high-protein haplotypes exhibited phenotypes with seed protein content exceeding 43.0% (Figure 4D). These excellent haplotypes and their pyramiding may provide new genetic resources for high-protein soybean breeding.

KASP markers, leveraging their inherent characteristics, enhance breeding accuracy and expedite the transfer and aggregation of genes, thus improving actual breeding efficiency. However, challenges arise when transitioning from hybrid populations to practical breeding scenarios. Intergenic effects, including epistatic and masking effects between genes, can influence marker effectiveness. Additionally, variations in environmental conditions and genetic backgrounds can also impact the performance of KASP markers [85]. In this study, the three newly developed KASP markers demonstrated good genotyping effects and phenotype prediction rates within the hybrid population, laying a foundation for their application in practical breeding. Moreover, these markers, located in the QTL for seed protein content, were consistently detected across different populations, environments, and years, suggesting their potential stability. Haplotype analysis further indicates that the candidate genes associated with these markers possess a dominant haplotype linked to seed protein content. These attributes are advantageous for the effective application of the three KASP markers across diverse genetic backgrounds. 

## 4. Materials and Methods

For the 102 candidate genes associated with seed protein content, sequence alignment was conducted using resequencing data from the high-protein variety SN76 and two low-protein varieties, DS1 and SN49, to identify SNPs within the CDS region of these genes. KASP markers were developed for these SNPs and were found to have good genotyping performance. Subsequently, two F_6_ populations were used to validate the predictive accuracy of the markers, with three KASP markers demonstrating high prediction accuracy (Figure 1). Haplotype analysis was then conducted on the candidate genes associated with these markers.

### 4.1. Plant Materials and Protein Content Profiling

The phenotypic prediction accuracy of KASP markers was rigorously validated using two F_6_ populations. These populations were developed from crosses between the high-protein-content cultivar Suinong 76 (SN76) and the lower-protein-content cultivars Suinong 49 (SN49) and Dongsheng 1 (DS1) (Table S4). The F_1_ generation was obtained by crossing SN49 and DS1 with SN76. This generation was then self-crossed to yield the F_2_ generation. Following a series of self-fertilization and selection, a recombinant inbred line population, comprising multiple strains, was established in the F_6_ generation for the prediction of seed protein content phenotypes (Figure S3). Throughout the 2021 and 2022 growing seasons, the experimental plots were established at the Suihua branch of the Heilongjiang Academy of Agricultural Sciences, China (46.61° N, 126.89° E). Each entry was represented in triplicate plots, with each plot comprising two rows measuring 5 m in length, arranged in a randomized block design and managed according to standard agricultural practices. Furthermore, the resequencing data of 643 soybean accessions, comprising 547 previously published by our laboratory [86] and an additional 96 newly collected varieties from Northeast China, were used to detect the haplotype of candidate genes and gene aggregation effects. The 643 soybean accessions were planted at the Xiangyang experimental station of Northeast Agricultural University, Harbin, Heilongjiang, China (45.75° N, 126.53° E) during the 2021 and 2022 growing seasons (Table S5). Post-harvest, all soybean plants were dried to achieve a moisture content below 13%. Subsequently, the seed protein content was determined using a near-infrared seed analyzer (Infratec 1241TM seed analyzer, Dresden, Germany). 

### 4.2. Molecular Marker Development and Genotyping

Genomic DNA was extracted from the two F_6_ populations and three parental lines by the cetyltrimethylammonium bromide (CTAB) method [87]. Sequence alignment and SNP positions were obtained by referencing the genome *Glycine max* (Wm82.a4.v1). For the SNP sites identified, we selected the flanking 50 base pair sequences, both upstream and downstream. The design of KASP primers was facilitated by Premier 5.0 software, comprising two specific forward primers and a common reverse primer. These forward primers are distinguished by their capacity to discern different alleles and are labeled with distinct fluorescent tags, FAM and HEX, at their 5’ ends for color-based differentiation of PCR products. Detailed primer sequence information for the three KASP markers can be found in Table S7. The PCR reactions were conducted using a Roche LightCycler 480 II (Basel, Switzerland), a real-time fluorescent quantitative PCR instrument. Each 10 µL reaction system contained 5 µL of DNA, 3.2 µL of master mix, 0.28 µL of primer, and 1.52 µL of ddH_2_O. The PCR protocol included an initial denaturation step at 95 °C for 30 s, followed by 10 touchdown cycles of 60 s at 95 °C and annealing for 20 s commencing at 63 °C with a decrement of 0.8 °C per cycle down to 55 °C. This was succeeded by 30 cycles of 60 s at 95 °C and 20 s at 55 °C and concluded with a final step at 37 °C for 60 s. The KASP genotyping results were interpreted and a genotyping map was generated using TaqMan Genotyper software version 1.7.1.

### 4.3. Determination of Prediction Accuracy

The predictive accuracy of the genetic markers was evaluated in accordance with the methodology described below. The high-protein predictive accuracy was calculated based on the proportion of strains that displayed a high-protein phenotype relative to those that contained the corresponding high-protein alleles. Similarly, the low-protein predictive accuracy was determined by contrasting the count of lines expressing a low-protein phenotype against the lines that had low-protein alleles. Moreover, the distinction between high- and low-protein groups is based on the average seed protein content plus or minus the standard deviation of the population. The comprehensive predictive accuracy was derived by averaging the accuracies from both high- and low-protein predictions.

### 4.4. Haplotype Analysis

Haplotypes of the candidate genes were meticulously examined within the 643 reported germplasm resource population using DNASP 5.10 software [88]. Phenotypic variances among the distinct haplotypes for each candidate gene of significance were meticulously evaluated based on the quantified seed protein content phenotype. Subsequently, the haplotypes were systematically categorized into high- and low-protein groups. 

### 4.5. Statistical Analysis

Phenotypic and genotypic data were organized using Microsoft Excel. Subsequently, SPSS version 17.0 was employed to perform a two-tailed *t*-test or multiple comparisons using Tukey’s honestly significant difference (HSD) test for statistical significance analysis.

## 5. Conclusions

Three KASP markers were developed based on our previous studies on meta-QTLs for seed protein content, and the candidate genes were analyzed using 643 accessions. Through allele identification, these markers were validated using two F_6_ populations. The effectiveness of these markers is further supported by the seed protein content and the high predictive accuracy of these results. Additionally, the genes associated with the KASP markers, *Glyma.03G219900*, *Glyma.14G119000*, and *Glyma.17G074400*, are potential key candidate genes for seed protein content. Haplotype analysis and gene pyramiding combinations were performed using germplasm resources, leading to the identification of several superior haplotypes and pyramiding combinations. These markers may provide new insights for molecular breeding of high-protein soybean. 

## Figures and Tables

**Table 1:** Summary of SNP information on the candidate genes associated with protein content.

SNP Number	Gene Name	Position (bp)	Condon Change	Amino Acid Change
1	*Glyma.02G090800*	7,982,533	G/A	Val > Met
2	*Glyma.02G090800*	7,983,388	A/G	Asn > Asp
3	*Glyma.02G274900*	47,624,912	T/A	Ser > Arg
4	*Glyma.02G274900*	47,625,653	T/C	Phe > Leu
5	*Glyma.03G219900*	43,490,795	T/A	Tyr > Asn
6	*Glyma.03G232000*	44,494,979	T/G	Ile > Met
7	*Glyma.07G051500*	4,424,891	T/G	Leu > Val
8	*Glyma.07G151300*	18,219,051	G/A	Gly > Asp
9	*Glyma.07G261900*	43,998,589	T/C	Tyr > His
10	*Glyma.07G261900*	43,998,608	T/C	Leu > Ser
11	*Glyma.12G018300*	1,280,719	A/G	Lys > Arg
12	*Glyma.12G230900*	40,525,414	G/A	Val > Ile
13	*Glyma.14G119000*	33,480,253	A/G	Ser > Gly
14	*Glyma.14G119000*	33,480,610	A/G	Thr > Ala
15	*Glyma.15G089800*	6,905,426	T/C	Leu > Pro
16	*Glyma.17G074400*	5,829,960	G/A	Cys > Tyr
17	*Glyma.17G074400*	5,830,014	T/C	Val > Ala
18	*Glyma.17G074400*	5,830,670	C/A	Thr > Lys
19	*Glyma.19G164800*	43,002,740	A/G	Asn > Asp

## Data Availability

All data are available in this manuscript or Supplementary Materials.

## Supplementary Materials

The following supporting information can be downloaded at https://www.mdpi.com/article/10.3390/plants13243485/s1. Figure S1: Fluorescence signal detection map labeled with three KASP markers; Figure S2: Allelic discrimination of hybrid populations using the KASP marker; Figure S3: Diagram of process for constructing two F_6_ populations; Table S1: Functional annotations of candidate genes related to seed protein content; Table S2: Candidate genes related to seed protein content with SNP differences in coding regions; Table S3. Protein content and amino acid composition of SN76, SN49, and DS1; Table S4: Phenotypic characteristics of two F_6_ populations in seed protein content; Table S5: Phenotypic characteristics of 643 accessions in seed protein content; Table S6: Reported QTL loci containing three candidate genes; Table S7. Primer sequence information of three KASP markers.
