# Supplementary material for: Development and Validation of Kompetitive Allele-Specific Polymerase Chain Reaction Markers for Seed Protein Content in Soybean
Source: Plants (Basel). 2024 Dec 13;13(24):3485. doi: 10.3390/plants13243485 (PMC11728539; doi:10.3390/plants13243485)
Supplement: Supplementary file 1 [file plants-13-03485-s001.zip › plants-3340791-supplementary.pdf]

Table S1. Functional annotations of candidate genes related to seed protein content.

Gene Model	Functional annotations
Glyma.01G106800	Heat shock protein 20 (HSP20)-like chaperone
Glyma.01G131100	Aspartate aminotransferase
Glyma.02G090800	Translation initiation factor IF2/IF5
Glyma.02G137200	Eukaryotic translation initiation factor 2A
Glyma.02G141300	Transmembrane amino acid transporter family protein
Glyma.02G151500	Protein SLOW WALKER 1
Glyma.02G205600	Heat shock 70 kDa protein-like
Glyma.02G268600	Vacuolar protein sorting-associated protein 29
Glyma.02G274900	Argonaute family protein
Glyma.03G129500	DNA-directed RNA polymerase II subunit family protein
Glyma.03G172200	Eukaryotic translation initiation factor 3 subunit A
Glyma.03G219900	DELLA protein
Glyma.03G232000	Probable protein disulfide-isomerase A6
Glyma.03G239700	Basic 7S globulin, BG7S-1
Glyma.03G244800	OAS-TL1, cysteine synthase
Glyma.04G052900	Eukaryotic initiation factor 4A-11
Glyma.04G094600	Argininosuccinate lyase
Glyma.04G122600	Elongation factor P (EF-P)- like family protein
Glyma.04G200700	Box C/D snoRNA 3'-end processing
Glyma.04G209200	Amino acid permease 3
Glyma.04G228000	Transparent testa glabra 1
Glyma.04G229800	Heat shock protein 21 (HSP21)
Glyma.04G242500	Probable indole-3-pyruvate monooxygenase
Glyma.05G046400	Nucleolar protein 56
Glyma.06G053200	Eukaryotic initiation factor 4A-2
Glyma.06G082500	Eukaryotic translation initiation factor 3 subunit L
Glyma.06G090200	Amino acid permease 6
Glyma.06G114000	No apical meristem (NAM), ATAF1/2, cup-shaped cotyledon2 (CUC2) NAC2
Glyma.06G120700	Probable indole-3-pyruvate monooxygenase
Glyma.06G136900	Transparent testa glabra 1
Glyma.06G142400	Aminoacyl tRNA synthase complex-interacting multifunctional protein 1
Glyma.06G197300	Eukaryotic translation initiation factor 5A-2
Glyma.06G227200	Transport protein Sec61 subunit alpha
Glyma.07G007400	Eukaryotic initiation factor 4A-10
Glyma.07G043600	Heat shock protein 20 (HSP20)-like chaperone protein
Glyma.07G051500	Transcription factor MYC2
Glyma.07G090900	Translation initiation factor, eIF-2B subunit beta
Glyma.07G102800	Vacuolar protein sorting-associated protein 26C
Glyma.07G107700	Eukaryotic translation initiation factor 3 subunit B
Glyma.07G151300	Fatty acid desaturase 8
Glyma.07G200300	Heat shock protein 20 (HSP20)-like chaperone
Glyma.07G200500	Heat shock protein 17.5-M

Table S1. Continued

Gene Model	Functional annotations
Glyma.07G261900	U3 small nucleolar RNA-associated protein 6 homolog
Glyma.08G052900	Eukaryotic translation initiation factor 3 subunit C2
Glyma.08G069000	Heat shock protein 17.3-B,17.3 kDa class I heat shock protein
Glyma.08G190100	Eukaryotic initiation factor 4A-10
Glyma.08G212000	Heat shock protein 21 (HSP21)
Glyma.08G316700	Protein translation factor SUI1 homolog 2
Glyma.09G000700	60S ribosomal protein L3
Glyma.09G018300	Vacuolar protein sorting-associated protein 53 A
Glyma.09G131500	Heat shock protein 90A1
Glyma.09G173200	GS1alpha, cytosolic glutamine synthetase alpha
Glyma.09G230700	DNA-directed RNA polymerase III subunit RPC4 isoform X1
Glyma.10G022000	Polycomb group protein FERTILIZATION-INDEPENDENT ENDOSPERM-like
Glyma.10G022600	Protein SLOW WALKER 1
Glyma.10G037100	Glycinin G4, GY4
Glyma.10G176400	Heat shock protein 20 (HSP20)-like
Glyma.10G225800	Eukaryotic translation initiation factor 5B
Glyma.10G246300	Beta-conglycinin alpha prime subunit, CG-1
Glyma.10G246500	Beta-conglycinin beta subunit 1-like
Glyma.11G032800	40S ribosomal protein S30
Glyma.11G134200	18.5 kDa class I heat shock protein
Glyma.11G154500	DNA-directed RNA polymerase V subunit 5C
Glyma.11G226000	Proline transporter 1
Glyma.12G002800	RNA-binding protein 24
Glyma.12G018300	Probable methionine-tRNA ligase
Glyma.12G156800	Transport protein Sec61 subunit alpha
Glyma.12G161500	Tyrosine aminotransferase 3
Glyma.12G230900	Transport protein Sec61 subunit alpha
Glyma.12G240600	60S ribosomal protein L3
Glyma.13G049700	Proline dehydrogenase 2
Glyma.13G062000	No apical meristem (NAM), ATAF1/2, cup-shaped cotyledon2 (CUC2) (NAC) Domain-containing protein 100
Glyma.13G106200	DEAD-box ATP-dependent RNA helicase
Glyma.13G115500	Alpha-aminoadipic semialdehyde synthase
Glyma.13G123500	Glycinin gy5, GY5
Glyma.13G171200	Ribosomal RNA-processing protein 7 homolog A
Glyma.13G176000	Heat shock protein 17.6-L, 17.6 kDa class I heat shock protein
Glyma.13G176200	Heat shock protein 17.5-E
Glyma.13G268600	Transport protein Sec61 subunit alpha-like
Glyma.14G048800	Vacuolar sorting protein
Glyma.14G102700	Chorismate mutase 2
Glyma.14G119000	Myeloblastosis (MYB) domain protein 56

Table S1. Continued

Gene Model	Functional annotations
Glyma.14G196700	NHP2-like protein 1
Glyma.15G026100	Eukaryotic initiation factor 4A-10
Glyma.15G042100	Glutelin type-A 1-like, 11-S seed storage protein
Glyma.15G089800	Eukaryotic translation initiation factor 4E-1
Glyma.16G018400	Vacuolar protein sorting-associated protein 8 homolog
Glyma.16G178800	Heat shock protein 90-A2
Glyma.17G010750	Leucine--tRNA ligase
Glyma.17G053200	DEAD-box ATP-dependent RNA helicase
Glyma.17G072400	Heat shock protein 70
Glyma.17G074400	Fatty acid desaturase 6, lipid metabolic process
Glyma.17G088200	U3 small nucleolar ribonucleoprotein protein MPP10
Glyma.17G103100	Eukaryotic translation initiation factor 5A-2
Glyma.17G192000	Omega-6 fatty acid desaturase
Glyma.17G231300	Phenylalanine--tRNA ligase alpha subunit
Glyma.19G042900	Proline dehydrogenase 2
Glyma.19G164800	Glycinin subunit G7, GY7
Glyma.19G236600	Basic 7S globulin 2, BG7S-2
Glyma.20G089400	Eukaryotic translation initiation factor 3 subunit M-like
Glyma.20G146200	Beta-conglycinin beta-subunit, CG-BETA-1
Glyma.20G148200	Beta-conglycinin, beta chain-like, CG-BETA-2

Table S2. Candidate genes related to seed protein content with SNP differences in coding region.

Gene Name	Annotation Description
Glyma.02G090800	Translation initiation factor IF2/IF5
Glyma.02G151500	Protein SLOW WALKER 1
Glyma.02G274900	Chromosome and associated proteins
Glyma.03G219900	DELLA protein
Glyma.03G232000	Probable protein disulfide-isomerase A6
Glyma.03G244800	OAS-TL1, cysteine synthase
Glyma.07G051500	Transcription factor MYC2
Glyma.07G102800	Vacuolar protein sorting-associated protein 26C
Glyma.07G151300	Omega-3 fatty acid desaturase
Glyma.07G261900	U3 small nucleolar RNA-associated protein 6
Glyma.08G069000	heat shock protein 17.3-B,17.3 kDa class I heat shock protein
Glyma.08G316700	Protein translation factor SUI1 homolog 2
Glyma.09G018300	Vacuolar protein sorting-associated protein 53 A
Glyma.09G230700	DNA-directed RNA polymerase III subunit RPC4 isoform X1
Glyma.10G037100	Glycinin G4, GY4
Glyma.12G018300	tRNA aminoacylation for protein translation
Glyma.12G230900	Transport protein Sec61 subunit alpha
Glyma.13G171200	Ribosomal RNA-processing protein 7 homolog A
Glyma.13G176000	heat shock protein 17.6-L, 17.6 kDa class I heat shock protein
Glyma.14G048800	Vacuolar sorting protein
Glyma.14G119000	Myeloblastosis (MYB) domain protein 56
Glyma.15G089800	Eukaryotic translation initiation factor 4E-1
Glyma.16G018400	Vacuolar protein sorting-associated protein 8 homolog
Glyma.16G178800	heat shock protein 90-A2
Glyma.17G074400	Omega-6 fatty acid desaturase
Glyma.19G164800	Glycinin subunit G7, GY7
Glyma.20G146200	Beta-conglycinin beta-subunit, CG-BETA-1

Table S3. Protein content and amino acids composition of SN76, SN49 and DS1.

Protein and Amino acids	SN76	SN49	DS1
Protein content (%)	46.65±0.31 a	42.45±0.05 b	41.41±0.07 c
Cys (%)	0.60±0.02 a	0.61±0.02 a	0.57±0.03 a
Phe (%)	2.28±0.03 a	2.15±0.04 b	2.06±0.05 c
Ala (%)	1.83±0.01 a	1.72±0.02 b	1.71±0.02 b
Met (%)	0.60±0.02 a	0.60±0.02 a	0.56±0.01 b
Gly (%)	1.85±0.02 a	1.74±0.02 b	1.70±0.02 c
Glu (%)	7.95±0.07 a	7.28±0.17 b	7.08±0.17 b
Arg (%)	3.37±0.06 a	2.98±0.05 b	2.98±0.09 b
Lys (%)	2.87±0.01 a	2.68±0.04 b	2.65±0.05 b
Tyr (%)	1.52±0.02 a	1.50±0.03 a	1.41±0.02 b
Leu (%)	3.39±0.02 a	3.11±0.06 b	3.06±0.07 b
Pro (%)	2.11±0.04 a	1.99±0.02 b	1.89±0.04 c
Trp (%)	0.42±0.02 b	0.46±0.01 a	0.41±0.02 b
Ser (%)	1.90±0.02 a	1.81±0.03 b	1.68±0.03 c
Thr (%)	1.65±0.02 a	1.57±0.03 b	1.51±0.02 c
Asp (%)	5.01±0.04 a	4.55±0.07 b	4.54±0.11 b
Val (%)	2.21±0.01 a	1.99±0.03 b	2.03±0.02 b
Ile (%)	2.10±0.02 a	1.94±0.03 b	1.95±0.03 b

Different lowercase letters indicate significant differences at the $P < 0.05$ level.

Table S4. Phenotypic characteristics of two F_6 population in seed protein content.

F_6 population	Year	Minimum	Maximum	Range	Median	Average	Standard deviation	Coefficient of variation
DS population	2021	32.36	43.47	11.11	39.86	39.49	1.95	4.94%
DS population	2022	33.42	44.1	10.68	40.54	40.17	2.16	5.37%
SS population	2021	31.53	46.63	15.1	40.65	40.49	2.4	5.92%
SS population	2022	31.03	45.93	14.9	40.33	40.2	2.1	5.53%

Table S5. Phenotypic characteristics of 643 accessions in seed protein content.

Trait	Year	Minimum	Maximum	Range	Median	Average	Standard deviation	Coefficient of variation
Seed protein content	2021	37.52	48.43	10.91	42.1	42.21	1.88	4.44
Seed protein content	2022	37.85	48.73	10.88	42.25	42.5	1.85	4.35

Table S6. Reported QTL loci containing three candidate genes.

Gene	SNP position	Trait	Combination	Markers	Chr.	Start_ position	End_ position	Reference
Glyma.03G219900	43,490,795	Seed protein content	Dongnong46 × Kenjian23	Sat_275, Satt022	Gm03	29,862,641	44,682,615	Mao et al. [59]
		Seed protein content	Dongnong46 × Kenjian23	Sat_295, Satt022	Gm03	40,375,902	44,682,615	Mao et al. [59]
		Seed yield	JP110755 × Fukuyutaka	Satt022, Satt234	Gm03	40,168,335	44,682,615	Kuroda et al. [60]
		Seed oil content	Charleston × Dongnong	Satt022, Satt257	Gm03	41,520,601	44,682,615	Qi et al. [61]
		Seed oil content	SD02-4-59 × A02-381100	Satt022	Gm03	42,675,829	44,682,615	Wang et al. [62]
		Seed oil content	Dongnong46 × Kenjian23	Sat_275, Satt022	Gm03	29,862,641	44,682,615	Mao et al. [59]
		Seed thickness	Bogao × Nannong94-156	Satt022, Satt339	Gm03	37,917,420	44,682,615	Jun et al. [63]
		Seed length	Noir1 × Minsoy	Satt022, Satt234	Gm03	40,168,335	44,682,615	Salas et al. [64]
Glyma.14G119000	33,480,253	Seed protein content	Dongnong46 × Kenjian23	Sat_189, Satt070	Gm14	16,352,945	33,180,365	Mao et al. [59]
		Seed protein content	Jindou23 × Huibuzhi	Satt063, Satt070	Gm14	17,406,101	46,705,840	Liang et al. [65]
		Seed protein content	Dongnong46 × Kenjian23	Satt070, Satt202	Gm14	17,406,101	48,441,504	Mao et al. [59]
		Seed yield	PI68658 × Lawrence	Satt474	Gm14	16,248,799	33,076,661	Fox et al. [66]
		Seed yield	Kenwood × LG94-1713	Satt474	Gm14	16,248,799	33,076,661	Guzman et al. [67]
		Seed oil content	Dongnong46 × Kenjian23	Sat_189, Satt063	Gm14	16,352,945	46,705,840	Mao et al. [59]
		Seed oil content	Jindou23 × Huibuzhi	Satt063, Satt070	Gm14	17,406,101	46,705,840	Liang et al. [65]
		Seed oil content	Dongnong46 × Kenjian23	Satt070, Satt202	Gm14	17,406,101	48,441,504	Mao et al. [59]
		Seed width	Bogao × Nannong94-156	Satt070, Satt355	Gm14	17,406,101	34,757,105	Jun et al. [63]
Glyma.17G074400	5,830,014	Seed oil content	Essex × Williams	Satt154, Satt458	Gm17	5,788,551	9,863,214	Hyten et al. [68]
		Seed weight	KefengNo1 × Nannong1138-2	Satt458	Gm17	5,788,551	6,053,036	Zhang et al. [69]
		Shoot weight	BD2 × BX10	Satt458	Gm17	5,788,551	6,053,036	Liang et al. [70]

Table S7. Primer sequence information of three KASP markers.

KASP Marker	Sequences	High protein allele	Low protein allele
KASP-Pro-1	FAM_primer: GAAGGTGACCAAGTTCATGCTAAGGCCTGATTGCATGGAGA<br>HEX_primer: GAAGGTCGGAGTCAACGGATTAAGGCCTGATTGCATGGAGT<br>Reverse primer: GGTTCACCAAGGAGGGTGAG	T	A
KASP-Pro-2	FAM_primer: GAAGGTGACCAAGTTCATGCTGAAAGGAACTTCTTTTTGTCCACTA<br>HEX_primer: GAAGGTCGGAGTCAACGGATTGAAAGGAACTTCTTTTTGTCCACTG<br>Reverse primer: CTTCTTCGTCAGTGCAAGTGC	A	G
KASP-Pro-3	FAM_primer: GAAGGTGACCAAGTTCATGCTCCCTGGTATCTTCTTCCTCTGGT<br>HEX_primer: GAAGGTCGGAGTCAACGGATTCCCTGGTATCTTCTTCCTCTGGC<br>Reverse primer: GTGTCGTCACTAAGAAATAATGATAAGG	T	C

The underlines represent the fluorescent tag FAM and HEX sequences.

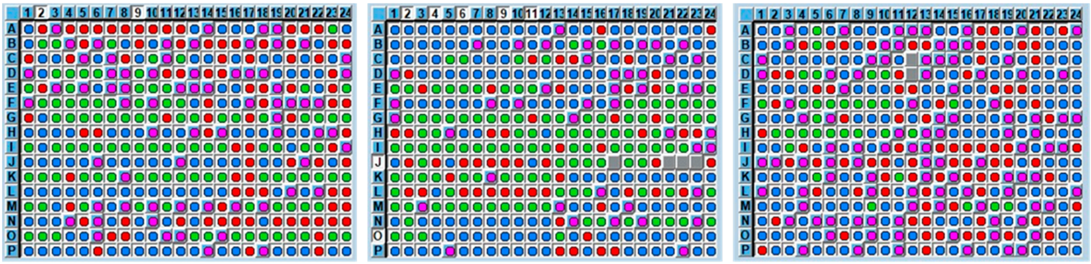

KASP-Pro-1 KASP-Pro-2 KASP-Pro-3

Figure S1. Fluorescence signal detection map labeled with three KASP marker. Each dot corresponds to an individual tested. Green or blue dots represent homozygous genotypes, red dots represent heterozygotes.

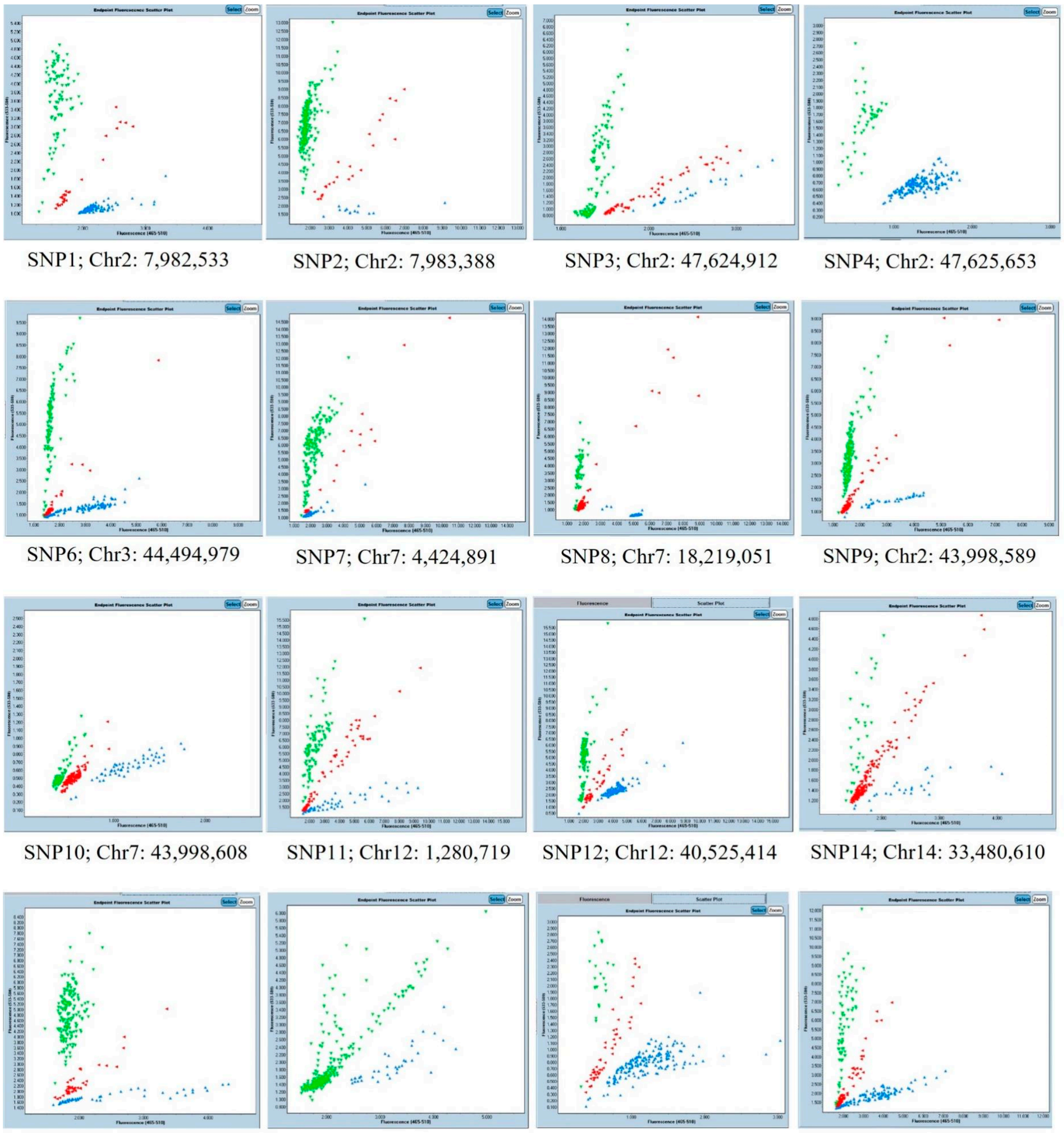

SNP1; Chr2: 7,982,533 | SNP2; Chr2: 7,983,388 | SNP3; Chr2: 47,624,912 | SNP4; Chr2: 47,625,653

SNP6; Chr3: 44,494,979 | SNP7; Chr7: 4,424,891 | SNP8; Chr7: 18,219,051 | SNP9; Chr2: 43,998,589

SNP10; Chr7: 43,998,608 | SNP11; Chr12: 1,280,719 | SNP12; Chr12: 40,525,414 | SNP14; Chr14: 33,480,610

SNP15; Chr15: 6,905426 | SNP16; Chr17: 5,829,960 | SNP18; Chr17: 5,830,670 | SNP19; Chr19: 43,002,740

Figure S2. Allelic discrimination of hybrid populations using the KASP marker. Each dot corresponds to an individual tested. Green or blue dots represent homozygous genotypes, red dots represent heterozygotes.

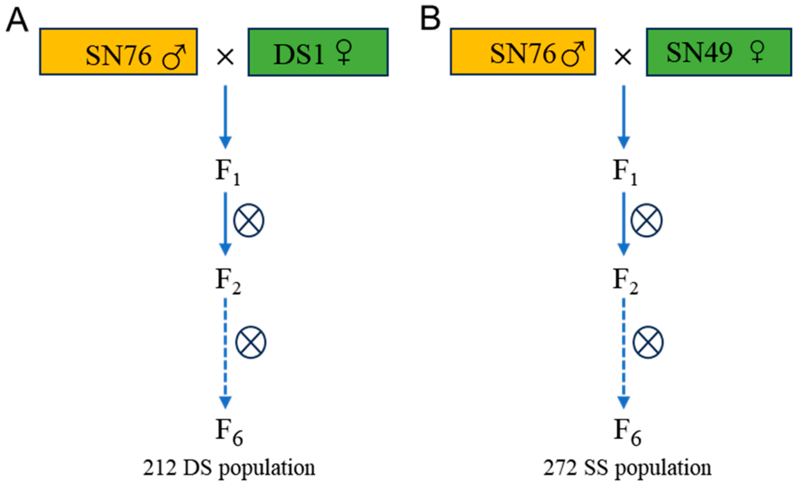

Figure S3. Diagram process for constructing two F_6 populations. A. DS population, B. SS population.
